# Smart Polymeric Wound Dressing for Treating Partial-Thickness Burns: A Preliminary Preclinical Study on the Porcine Model

**DOI:** 10.3390/ebj4010004

**Published:** 2023-01-15

**Authors:** Dmitry Beylin, Josef Haik, Erik Biros, Rachel Kornhaber, Michelle Cleary, Moti Harats, Daniel Cohn, Yair Sapir, Ori Weisberg

**Affiliations:** 1 The National Burn Center, Sheba Medical Center, Tel Hashomer, Ramat Gan 52621, Israel; 2 Sackler Faculty of Medicine, Tel-Aviv University, Tel-Aviv-Yafo 69978, Israel; 3 Institute for Health Research, University of Notre Dame, Fremantle, WA 6959, Australia; 4 The Talpiot Medical Leadership Program, Sheba Medical Center, Ramat Gan 52621, Israel; 5 College of Health and Medicine, University of Tasmania, Sydney, NSW 2015, Australia; 6 College of Medicine & Dentistry, James Cook University, Townsville, QLD 4811, Australia; 7 School of Nursing, Midwifery & Social Sciences, CQ University, Sydney, NSW 2000, Australia; 8 Casali Center of Applied Chemistry, Institute of Chemistry, Hebrew University of Jerusalem, Jerusalem 9190401, Israel; 9 Inteligels Ltd., Hoshaya 1791500, Israel

**Keywords:** burns, partial thickness, dressings, smart bandage, porcine model

## Abstract

Several so-called “smart” dressings are available for burn injuries to promote faster wound healing, and this technology has recently reported substantial advancements. However, the selection of an appropriate dressing for partial-thickness burns requires consideration of several crucial elements, including exudate management, conformability, antimicrobial properties, ease of application and removal, patient comfort, and cost-effectiveness. This preliminary feasibility study uses a porcine model to test the INTELIGELS product (Smart Bandage) for partial-thickness burns treatment. Artificially made wounds, mimicking partial-thickness burns, were assessed in two studies with and without antimicrobial additives, where wounds were randomly assigned to the experimental group treated with Smart Bandage and two control groups treated with a simple saline gauze dressing or Aquacel^®^ products with and without silver additives. In addition, all dressings were evaluated for their ability to reduce wound size, quantified by histological analysis using punch biopsies. This study demonstrates comparable healing properties of Smart Bandage and Aquacel^®^ dressings that are superior to the simple saline gauze dressing. The superiority is demonstrated by better regeneration, less inflammation of the epidermis and dermis, and better dermis remodeling with more granulation tissue maturation within the wound area when Smart Bandage/Aquacel^®^ dressings are applied as compared with the simple gauze dressing.

## 1. Introduction

According to the World Health Organization [1], burns represent a worldwide public health concern, accounting for approximately 180,000 deaths annually. Furthermore, burn injuries are associated with excruciating pain and prolonged hospitalizations, thus negatively impacting patients’ quality of life (QoL) [2]. Hence, ineffective burn wound treatment has significant financial and psychological repercussions and long-term somatic sequelae, exerting a significant burden on the healthcare system, patients, and their families. Therefore, it is crucial to develop appropriate and effective burn wound therapies. In recent years, the growing interest and advancements in tissue engineering and biomaterials served as a technological scaffold, developing new approaches to treating cutaneous wounds that have lost their ability to heal spontaneously [3]. As a result, burn wound dressings are widely used to alleviate pain, decrease burn-related complications, prevent fluid loss and infection, and hasten the healing process [4]. While there are well-defined management protocols for superficial and full-thickness burns, partial-thickness burns remain challenging, and optimal therapy for these partial-thickness burns remains to be established [5].

There are currently over 3000 dressings available on the market, providing a wide range of solutions addressing different aspects and needs of wound care, including burn wounds [6]. These dressings include semipermeable silicone foams, tissue engineering scaffolds, hydrofibers, hydrogels, and hydrocolloid dressings. Hydrocolloids, in particular, have generated interest due to their unique benefits in treating burns, including their role in first aid where there is a lack of clean water, hydrating the wound, absorbing exudate, and activated autolytic debridement of the devitalized tissues [7]. Furthermore, while keeping the wound bed from drying, hydrocolloids also facilitate tissue repair and cell migration, providing access to vital nutrients for cell metabolism by allowing the penetration of immunological and growth factors, promoting the separation of necrotic/damaged tissue in a process called autolysis [8]. Furthermore, hydrocolloid dressings are easily applied, conform to the wound bed, provide highly customizable wound-sealing protection, and have been successfully used to treat chronic non-healing wounds [9].

A novel hydrocolloid dressing was recently developed [10,11,12] by INTELIGELS, referred to as the Smart Bandage, due to its programmable response and ability to carry active molecules that improve wound healing. The Smart Bandage (INTELIGELS Ltd. Hoshaya, Israel) is atraumatic, translucent, and provides comfortable wound protection that promotes rapid wound healing at a much lower cost and questions the necessity of the previously established traumatic wet-to-dry technique often unintentionally used with other dressings, without any debridement indicated. In this study, we elaborate on experiments conducted to test the feasibility of Smart Bandages developed by INTELIGELS to manage acute partial-thickness burn wounds and compare its healing properties with traditional simple saline gauze and a hydrofiber-type dressing (Aquacel^®^) using the porcine model.

## 2. Materials and Methods

Two studies were performed investigating the feasibility of Smart Bandages (INTELIGELS Ltd. Hoshaya, Israel) with and without antimicrobial additives for treating partial-thickness burns.

Study 1—Comparison of Smart Bandage (INTELIGELS Ltd. Hoshaya, Israel) without an antimicrobial additive with Aquacel^®^ (ConvaTec, Reading, UK) and simple saline gauze dressings.

### 2.1. Handling of Animal, Anesthesia, and Ethical Considerations

Israeli laws and regulations were followed to ensure animal welfare and strict experimental procedures. In particular, animal housing and care conditions were maintained according to institutional guidelines and approved by the Institutional Committee on Animal Use (ethical approval number IL0500321). The study used a 70-kg healthy, pink unicolor female pig (*Sus domesticus*) housed in an individual pen in a room with an artificial 12-h light/dark cycle and fed ad libitum with laboratory chow and water. The animal housing facility is positioned at the B. Rappaport Faculty of Medicine, Technion, Israel Institute of Technology, Israel.

The burn wounds and subsequent dressing changes were conducted under anesthesia in a non-sterile environment but using sterile instruments and techniques to reduce contamination. The pig was anesthetized using the following protocol: premedication with Ketamine 20 mg/kg and Xylazine 1 mg/kg intramuscular. Induction was performed using Propofol 3–5 mg/kg intravenously. Isoflurane 1–3% delivered with 100% O_2_ for maintenance via a ventilator. Postoperatively, Ceforal (Cephalexin) 1 g twice daily and Buprenorphine (Analgesia) 0.03 mg/kg body weight twice daily for three days. In addition, the pig received standard care and Fentanyl 3–5 mcg/kg/h for analgesia intravenously constant rate infusion based on veterinary recommendations. The pig was assessed daily throughout the experiment by trained veterinary personnel for signs of pain or distress, such as reduced activity, altered overall appearance, changes in temperament, vocalizations, and food and water consumption. In addition, once a week, the pig underwent a physical examination. The pig did not appear to experience unusual pain or require any additional medical intervention besides the one used according to the protocol. At the end of the experiment, pigs were euthanized using Isoflurane 5%, and Pental was given at the rate of 10 mg/kg/h.

### 2.2. Creation of the Partial-Thickness Burn

The pig’s skin was prepared by shaving using a razor and disinfected with septal scrub prior to creating the burns. Along the dorsal spine in four rows, the positioning of the burn wounds was marked with a sterile permanent marker prior to burning. A total of 16 partial-thickness burn wounds (Smart Bandage (INTELIGELS Ltd. Hoshaya, Israel), simple saline gauze, and Aquacel^®^ dressing groups, *n* = 5 each and one control) were created 4 cm in diameter and 5 cm apart using a cylindrical brass rod weighing 358 g placed in hot water (92 °C) for 2 min and then placed on the pig’s dorsal skin for 20 s with no additional pressure, producing partial-thickness burns.

### 2.3. Dressing

Three dressings were studied: Smart Bandage (INTELIGELS Ltd. Hoshaya, Israel), Aquacel^®^ (ConvaTec), and a wet-to-dry dressing consisting of simple saline gauzes. Each group of the five partial-thickness burn wounds was randomly assigned to be treated with one of the dressings. Dressings were replaced on days 3, 7, 11, and 14 of the 17-day study period for photography, biopsy, and documentation purposes, and the wounds were irrigated with normal saline. In addition, dressings were covered with a non-adherent gauze pad, which was kept in place using Tensoplast Elastic Adhesive Bandage (Smith & Nephew, Kingston upon Hull, United Kingdom) to prevent removal by the animal. On each treatment day, the pig was weighed, and each burn wound was photographed using a 12-megapixel digital camera (Nikon, Tokyo, Japan).

### 2.4. Analysis

Punch biopsy specimens from the center of three wounds of each group were collected on day 17 using an 8-mm circular blade. The specimens were immediately fixed in 4% neutral buffered formalin for 24 h and then transferred to 70% ethanol for histological analysis.

### 2.5. Histological Analysis

Histological scoring was performed blindly by one independent observer. Scoring of the histological parameters (Table A1 and Table A2) is demonstrated in Table A3. The parameters evaluated are regeneration of the epidermis (epithelialization), inflammation on the skin surface (either pustule formation or serocellular crusting), inflammation of the dermis and the subcutaneous adipose panniculus (scoring of leukocytic infiltration in the dermis and or in the subcutaneous fat), presence and maturation of granulation tissue in the area of the wound (remodeling of the dermis), and appearance of the dermis (normal adnexa or decreased adnexal numbers due to scarring or necrosis). Scoring is semiquantitative. For easier reading and interpretation of the score, 0 indicates normal tissue, and 4 indicates an active/severe lesion. In addition to these parameters, a macroscopical assessment of wound appearance was evaluated by independent, experienced clinicians within the wound regeneration field.

Study 2—Comparison of Smart Bandage+ (INTELIGELS Ltd. Hoshaya, Israel), a Smart Bandage with added antimicrobial agent polyhexamethylene biguanide (PHMB), with Aquacel^®^ Ag (Aquacel with a silver additive; ConvaTec), and simple saline gauze dressings.

This second study aimed to check the safety and feasibility of combining the Smart Bandage (INTELIGELS Ltd. Hoshaya, Israel) with PHMB. For this initial study, we used a 70-kg healthy pink unicolor female pig (*Sus domesticus*), and animal handling and housing were performed as described above for Study 1. In brief, burn wounds and subsequent dressing changes were conducted under anesthesia in a nonsterile environment but using sterile instruments and techniques to reduce contamination. The pig was anesthetized using the following protocol: premedication with Ketamine 20 mg/kg and Xylazine 1 mg/kg intramuscular. Induction was performed using Propofol 3–5 mg/kg intravenously. Isoflurane 1–3% delivered with 100% O_2_ for maintenance via a ventilator. Postoperatively, Ceforal (Cephalexin) 1 g twice daily and Buprenorphine (Analgesia) 0.03 mg/kg body weight twice daily for three days. In addition, the pig received standard care and Fentanyl 3–5 mcg/kg/h for analgesia intravenously constant rate infusion based on veterinary recommendations. The pig was assessed daily throughout the experiment by trained veterinary personnel for signs of pain or distress, such as reduced activity, altered overall appearance, changes in temperament, vocalizations, and food and water consumption. In addition, once a week, the pig underwent a physical examination. The pig did not appear to experience unusual pain or require any additional medical intervention besides the one used according to the protocol. At the end of the experiment, the pig was euthanized using Isoflurane 5%, and Pental was given at the rate of 10 mg/kg/h.

### 2.6. Creation of the Partial-Thickness Burn

The pig’s skin was prepared by shaving using a razor and disinfected with septal scrub prior to creating the burns. Along the dorsal spine in four rows, the positioning of the burn wounds was marked with a sterile permanent marker prior to burning. A total of 18 partial-thickness burn wounds were created for this study, each 4 cm in diameter and 5 cm apart using a cylindrical brass rod weighing 358 g, placed in hot water (100 °C) for 4 min, and then placed on the pig’s dorsal skin for 30 s with no additional pressure, producing partial-thickness burns.

### 2.7. Dressing

Wounds were treated with the Smart Bandage+ (INTELIGELS Ltd. Hoshaya, Israel) dressing, representing Smart Bandage (INTELIGELS Ltd. Hoshaya, Israel) with the addition of the antimicrobial agent polyhexamethylene biguanide (PHMB); an antimicrobial polymer that was mixed with the Smart Bandage (INTELIGELS Ltd. Hoshaya, Israel) polymer wound dressing at 0.02% by weight (Sinotrust International Trade Co. Ltd. Zhongshan Dist Dalian China). Three control groups were used: a simple saline gauze, Aquacel Ag^®^ (ConvaTec) containing silver as an antimicrobial agent, and one control without any treatment. Partial-thickness burn wounds were randomly assigned to the treatment group (Smart Bandage+ (INTELIGELS Ltd. Hoshaya, Israel); *n* = 9 wounds) or control groups, i.e., Aquacel Ag^®^ (*n* = 5 wounds), simple saline gauze (*n* = 4 wounds) with an emphasis on the treatment group to get better statistics for the safety of the new dressing. Dressings were replaced on days 3, 7, 10, and 13 of the 21-day study period for photography, biopsy, and documentation purposes. The wounds were irrigated with normal saline. In addition, dressings were covered with a non-adherent gauze pad, which was kept in place using Tensoplast Elastic Adhesive Bandage (Smith & Nephew) to prevent removal by the animal. On each treatment day, the pig was weighed, and each burn wound was photographed using a 12-megapixel digital camera (Nikon) with approximately the same angle, distance, and light conditions. In addition, Smart Bandage+ (INTELIGELS Ltd. Hoshaya, Israel) was also tested in vitro to determine its antibacterial efficacy against *Pseudomonas aeruginosa* and *Staphylococcus aureus*.

### 2.8. Microbiology

Single suspensions of *Pseudomonas aeruginosa* and *Staphylococcus aureus* (modified AATCC Test Method 100) were prepared to approximately 1 × 10^6^ mL^−1^ colony forming unit (CFU) in TSBTX-100. One milliliter of each bacterial suspension was used to inoculate control and test dressings that had been preconditioned according to protocol. Negative control dressing samples were immediately placed into 10 mL Quench solution according to protocol and sonicated to recover microorganisms from the dressing. Test dressing samples were incubated for 24 h at 37 ± 2 °C following the 24-h incubation period. The results were assessed statistically. Average Log_10_CFU per sample bacterial recoveries and average Log_10_CFU per sample reductions compared to the negative control were presented as mean ± standard deviation (SD). The minimum limit of detection for this study was 1.08 Log. A Student’s unpaired *t*-test (two-tailed) was used to assess statistical differences between the Log_10_CFU per sample recovery data from the negative control and test samples. Data were considered statistically significant when *p* < 0.05.

## 3. Results

Study 1—Assessment of Smart Bandage (INTELIGELS), Aquacel^®^ (ConvaTec), and a wet-to-dry dressing consisting of simple saline gauzes.

### 3.1. Macroscopic Assessment

**Figure 1:**
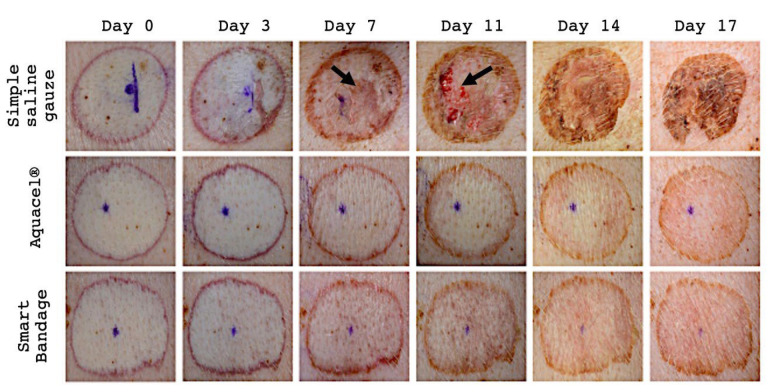
Representative image of burn (out of total *n* = 6) wounds treated with simple saline gauze, Aquacel^®^, Smart Bandage (INTELIGELS Ltd. Hoshaya, Israel) over a 17-day experimental period. The healing process in simple saline gauze areas demonstrates delayed healing, resulting in significant crusting. Arrows depict significant crusting on day seven and newly generated dermis damage on day 11 when simple saline gauze is used. While in both the Aquacel^®^ and Smart Bandage (INTELIGELS Ltd. Hoshaya, Israel), the healing process seems to demonstrate similar results.

A total of six burn wounds were analyzed, with two wounds in each group (Aquacel^®^, Smart Bandage (INTELIGELS Ltd. Hoshaya, Israel), and simple saline gauze dressing group); all wounds were made on one animal. All burns were photographed multiple times to visually inspect the progress of the wound healing process (Figure 1). The Aquacel^®^ and Smart Bandage (INTELIGELS Ltd. Hoshaya, Israel) dressings provide similar healing outcomes with minimal visible crusting and almost complete epidermal regeneration on day 17 (Figure 1). However, in simple saline gauze-treated burns, there is significant damage. The healing process is retarded, resulting in significant crusting visible on day 7, delaying the epidermal regeneration, and a much higher risk of infection due to the newly generated dermis damage (Figure 1).

### 3.2. Histology

**Figure 2:**
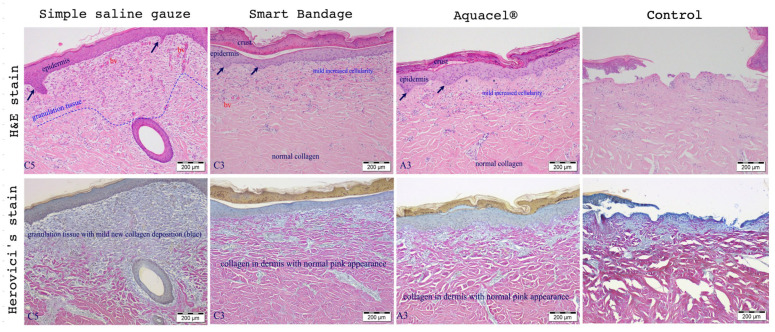
Histological representative wounds (out of a total *n* = 6) were evaluated on day 17 and treated with simple saline gauze, Smart Bandage (INTELIGELS Ltd. Hoshaya, Israel), and Aquacel^®^. Hematoxylin, eosin (H&E), and Herovici’s stains were used to evaluate wound healing and reactive fibrosis. Simple saline gauze showed less favorable results, while Smart Bandage (INTELIGELS Ltd. Hoshaya, Israel) and Aquacel^®^ perform very similarly and favor wound healing. Simple saline gauze increased fibrosis compared to the Smart Bandage (INTELIGELS Ltd. Hoshaya, Israel) and Aquacel^®^. Arrows indicate epidermal hyperplasia and the formation of rete pegs, which appear slightly increased in the simple saline gauze. The simple saline gauze dressing is associated with increased granulation tissue/fibrosis compared to the Aquacel^®^/Smart Bandage (INTELIGELS Ltd. Hoshaya, Israel). Control represents a partial-thickness burn done at day 0 and taken to histological evaluation. There is thermal necrosis of the epidermis and dermis and a focal sloughing of the necrotic epidermis; no evidence of regeneration or inflammation is evident.

**Figure 3:**
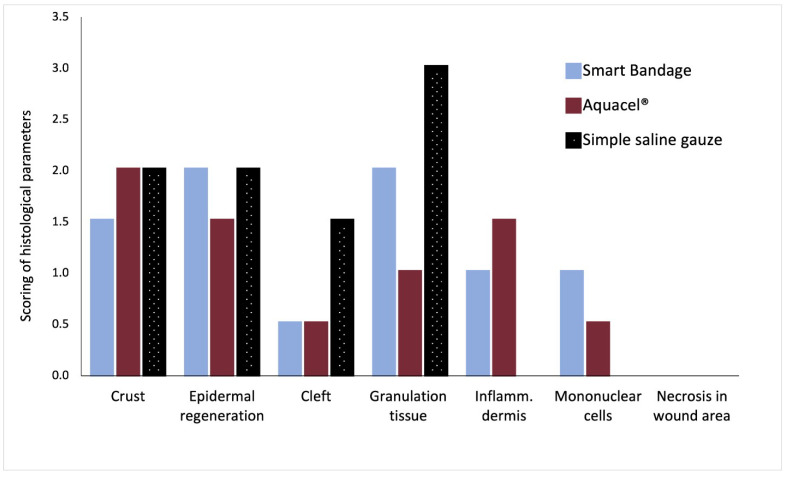
Scoring of tissue healing parameters by an independent pathologist. A total average score of *n* = 6 analyzed burns; Smart Bandage (INTELIGELS Ltd. Hoshaya, Israel) = 8, Aquacel^®^ = 7, simple saline gauze = 8.5 (see Appendix A Table A3).

Figure 2 shows the standard Hematoxylin and Eosin (upper row of Figure 2) and Herovici’s stain (bottom row of Figure 2) of burn wounds on day 17 post-injury treated with the Aquacel^®^, Smart Bandage (INTELIGELS Ltd. Hoshaya, Israel), and simple saline gauze dressings. Overall, Aquacel^®^ and Smart Bandage (INTELIGELS Ltd. Hoshaya, Israel) produce similar and favorable healing outcomes, while there is increased granulation tissue in the saline gauze sample (Herovici’s stain; bottom row of Figure 2). In addition, a histological evaluation of burn wounds on day 17 post-injury also identified a more prominent formation of dermo-epidermal clefting and granulation tissue when the simple saline gauze dressing was used to treat burns compared with the Aquacel^®^ or Smart Bandage (INTELIGELS Ltd. Hoshaya, Israel) dressings, as reflected in histological scoring (Figure 3).

Study 2–- Smart Bandage (INTELIGELS Ltd. Hoshaya, Israel) with the addition of polyhexamethylene biguanide dressing (PHMB).

### 3.3. Macroscopic Assessment

**Figure 4:**
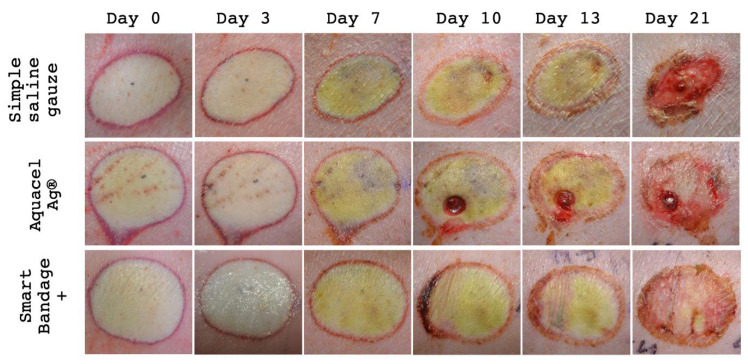
Representative burn wounds (out of total *n* = 18) treated with simple saline gauze, Aquacel Ag^®^, Smart Bandage+ (INTELIGELS Ltd. Hoshaya, Israel) (with the addition of PHMB) over 21 days. Clinically, in both Aquacel Ag^®^ and Smart Bandage+ (INTELIGELS Ltd. Hoshaya, Israel) dressings, the healing processes were very similar on day 21 (punch biopsy was taken from Aquacel Ag^®^ on day seven after the photo was taken). However, on day ten, a semilunar crust developed on the left edge of the wound, treated with Smart Bandage+ (INTELIGELS Ltd. Hoshaya, Israel). This was related to the manual removal of the dressing material and did not appear on the other samples. In addition, the wound treated with simple saline gauze demonstrates delayed healing with remnants of deep burn areas at the center on day 21 and crusting on the inferior site.

All burns were photographed multiple times to visually inspect the wound healing process (Figure 4). The Aquacel Ag^®^, Smart Bandage+ (INTELIGELS Ltd. Hoshaya, Israel) (with the addition of PHMB) dressings provide similar healing outcomes with minimal visible crusting and almost complete epidermal regeneration on day 21 (Figure 4). However, the wound treated with simple saline gauze demonstrates remnants of deep areas at the center on day 21 (Figure 4).

### 3.4. Histology

**Figure 5:**
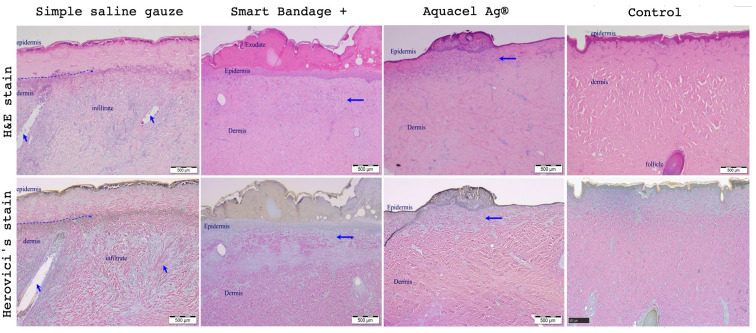
Histological representative wounds (out of total *n* = 18) evaluation on day 21, end of the study. Simple saline gauze, Smart Bandage+ (INTELIGELS Ltd. Hoshaya, Israel), and Aquacel^®^ treated wounds. Hematoxylin, Eosin, and Herovici’s stains were used to evaluate wound healing and reactive fibrosis. In the simple saline gauze samples, the blue dotted line on the left indicates the area of persistent epidermal and superficial dermal necrosis. The arrows indicate two necrotic hair follicles. There is underlying diffuse dermal cellular infiltration. The tissue is viable in the Smart Bandage+ (INTELIGELS Ltd. Hoshaya, Israel) and Aquacel Ag^®^ samples. As a result, there is epidermal regeneration and epidermal hyperplasia. The horizontal blue arrow on the right indicates the area of superficial dermal fibrosis. In the Smart Bandage+ (INTELIGELS Ltd. Hoshaya, Israel) sample, there are two necrotic follicles on both left and right margins of the photo. The control slide made at day 0 represents a complete thermal necrosis of the epidermis; the underlying hair follicle is viable. There was necrosis of the more superficial adnexa. There is no sloughing of the epidermis, inflammation, or regeneration attempts.

Figure 5 shows the standard Hematoxylin and Eosin (upper row) and Herovici’s stain (bottom row) stains of burn wounds on day 21 post-injury treated with the Aquacel Ag^®^, Smart Bandage+ (INTELIGELS Ltd. Hoshaya, Israel) (with the addition of PHMB) INTELIGELS, and simple saline gauze dressings. After the burn creation, marked thermal necrosis of the epidermis with necrosis of the underlying dermis, varying in depth from approximately 10% of the dermis to approximately 30%, thermal necrosis of hair follicles, and variable inflammation can be observed.

**Figure 6:**
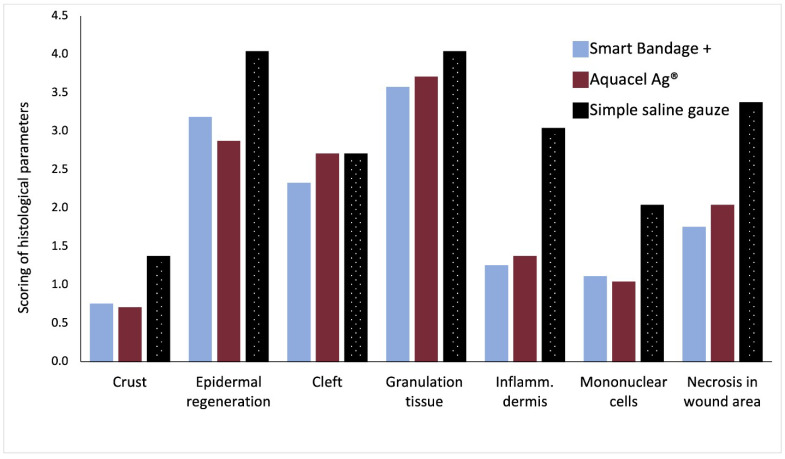
Scoring of tissue healing parameters by an independent pathologist. A total score of *n* = 6 analyzed burns; Aquacel Ag^®^ = 14.2, Smart Bandage+ (INTELIGELS Ltd. Hoshaya, Israel) = 13.7, and Simple saline gauze = 20.3 (see Appendix A Table A3).

Overall, Aquacel Ag^®^ and Smart Bandage+ (INTELIGELS Ltd. Hoshaya, Israel) produce similar and favorable healing outcomes, while the healing process is again impaired and underlined with markedly increased collagen production (best seen in Herovici’s stain; Figure 5) when the simple saline gauze dressing is used. In addition, a histological evaluation of burn wounds on each assessed day post-injury consistently showed favorable healing properties as evidenced by the lower scoring of parameters Aquacel Ag^®^, Smart Bandage+ (INTELIGELS Ltd. Hoshaya, Israel) (with the addition of PHMB) INTELIGELS as compared with the use of the simple saline gauze dressing (Figure 6).

The wounds were treated with Aquacel Ag^®^, Smart Bandage+ (INTELIGELS Ltd. Hoshaya, Israel), and simple saline gauze dressings. Hematoxylin and Eosin and Herovici’s stains were used to estimate epidermal regeneration and other healing process parameters. In simple saline gauze, the blue arrows indicate the lost adnexa. The intermittent blue line demarcates the area of necrosis in the epidermis and dermis. Smart Bandage+ (INTELIGELS Ltd. Hoshaya, Israel) and Aquacel Ag^®^ similarly demonstrate some degree of epidermal regeneration with hyperplasia, proteinaceous exudate, and superficial dermal fibrosis (arrow), as well as pale blue staining, indicating new collagen deposition (arrow in Herovici’s stain bottom row). It is seen that Aquacel Ag^®^ and Smart Bandage+ (INTELIGELS Ltd. Hoshaya, Israel) perform very similarly, while simple saline gauze impairs the healing process.

### 3.5. Microbiology

A microbiological assessment (Perfectus Biomed Ltd. Cheshire, UK) was performed in order to determine the antibacterial efficacy of Smart Bandage+ (INTELIGELS Ltd. Hoshaya, Israel) (with the addition of PHMB) versus Smart Bandage (INTELIGELS Ltd. Hoshaya, Israel) (negative control) against Gram-positive and Gram-negative bacteria, namely *Pseudomonas aeruginosa* and *Staphylococcus aureus*. These experiments were performed in vitro.

#### 3.5.1. Pseudomonas Aeruginosa

At 0 h, the negative control dressing observed an average viable *P. aeruginosa* recovery of 6.30 ± 0.03 Log_10_CFU per sample. Following 24 h of treatment, an average *P. aeruginosa* recovery of 6.11 ± 0.44 Log_10_CFU per sample was observed from Smart Bandage (INTELIGELS Ltd. Hoshaya, Israel) (without PHMB). No significant reduction or growth of *P. aeruginosa* was observed following treatment for 24 h with Smart Bandage+ (INTELIGELS Ltd. Hoshaya, Israel) (with PHMB additive) (Table 1).

#### 3.5.2. Staphylococcus Aureus

At 0 h, the negative control dressing observed an average viable S. aureus recovery of 6.01 ± 0.11 Log_10_CFU per sample. However, no viable *S. aureus* was recovered following 24 h of treatment with Smart Bandage+ (INTELIGELS Ltd. Hoshaya, Israel). This finding equated to a reduction of 6.01 ± 0.00 Log_10_CFU per sample (*p* < 0.001) when compared to the negative control at 0 h (Table 1).

Average recovery and a reduction in the quantity of viable *Pseudomonas aeruginosa* and *Staphylococcus aureus* recovered from test dressing samples compared to the negative control at 0 h. CFU = colony forming units, Log_10_ reduction per sample = average (Log_10_ CFU recovered from negative control at 0 h Log_10_ CFU recovered per dressing sample at 24 h), N/A = not applicable, SD = standard deviation.

## 4. Discussion

Occlusive dressings for burns and chronic wounds provide shielding from external infections and an essential evaporation barrier to promote wound healing [13]. In addition, numerous cell types and mediators, including cytokines, growth factors, proteolytic enzymes, and extracellular matrix components, are involved in restoring skin integrity as part of the burn healing process [13,14]. However, faster and more cost-effective clinical management within the burn intensive care units is needed due to the complexity of these partial-thickness burns and associated difficulties in their treatment. These persistent clinical needs, together with technological advances, prompted, over the last 30 years but precisely the last decade, biotech companies to invest in developing various biologically and biochemically functional wound dressings that can serve as dermal regeneration scaffolds or even incorporate living cells [6,14,15,16,17].

Winter’s [18,19] paradigm of wound care, published in the early 1960s, stated that wounds need to dry out to facilitate healing. This narrative has since shifted towards the importance of wound exudate management in the healing process with no risk of dressing-related infections [20,21]. However, if the dressing products/therapy of choice does not appropriately manage the wound exudate, this often increases the risk of infection [22]. The latter gave rise to the modern wound care practice and the increasing interest in bioactive dressings, including first-aid dressings, where much controversy emerged [23].

Among the various types of dressings, hydrocolloids have generated interest within the burn care discipline due to their unique properties in a tailored chemical environment, i.e., high porosity, facilitates the absorption of wound exudates yet maintains the delicate balance of hydration and has excellent permeability to the dry environment surrounding the wound [24]. In addition, hydrocolloids potentially encourage the autolytic debridement of the devitalized tissues, facilitating the healing process and prolonging the time between dressing changes [7]. Nevertheless, regardless of the advantages of these types of dressings, for nearly 50 years, silver-containing agents, namely silver sulfadiazine and hydrofiber, such as Aquacel Ag^®^, were conservatively used as an additive against bacterial colonization of wounds and partial-thickness burns [25,26,27,28,29,30,31]. However, in the last decade, there has been growing evidence suggesting the replacement of silver additives (Ag) with alternatives, such as polyhexamethylene biguanide (PHMB) [32,33,34,35,36]. Here, we aimed to test the feasibility of hydrocolloid products, the Smart Bandage (INTELIGELS Ltd. Hoshaya, Israel), and Smart Bandage with PHMB, known as Smart Bandage+, on artificially made partial-thickness burns in vivo; a type of burn that is known for a complicated healing process.

The Smart Bandage (INTELIGELS Ltd. Hoshaya, Israel) technology allows the clinician to easily apply the product on the wound bed/burn, solidifying and creating substantial barriers against the outside environment while maintaining transparency. Furthermore, the polymer liquifies again after applying the cleaning solution, making it easier for the medical staff to remove the dressing from the wound without retarding the healing process or causing discomfort to the patient. Another advantage of Smart Bandage (INTELIGELS Ltd. Hoshaya, Israel) technology worth considering is its cost-effectiveness.

We tested the Smart Bandage (INTELIGELS Ltd. Hoshaya, Israel) technology to determine whether it can improve wound healing using microscopic and macroscopic histological assessments. According to histological analysis of the burned sites at day 0, complete thermal necrosis of the epidermis was present, and no regeneration attempts were noted. However, at the end of the study, Smart Bandage (INTELIGELS Ltd. Hoshaya, Israel) demonstrated speedy healing comparable to traditional Aquacel^®^ dressing without any crusting and hyperplastic dermis, as noticed when simple saline gauze was used. Similar histological superiority was obtained when SmartBandage+ (with PHMB) and hydrofiber with silver additives (Aquacel Ag^®^) were used compared with simple saline gauze. In addition, observed regeneration of the epidermis and epidermal hyperplasia with granulation tissue formation in the superficial dermis (scarring) with minimal leukocyte infiltration suggests an inflammatory response presumably due to PHMB and Ag additives used in INTELIGELS and Aquacel^®^ dressings, respectively. However, further immunological-oriented studies are indicated to examine the resident wound leukocyte community when PHMB additive is used. We, however, tested the antimicrobial properties of PHMB in vitro to inform on potential aid with wound chronicity problems associated with microbial infections. We found that PHMB was associated with a marked reduction of S. aureus growth; however, *P. aeruginosa* seems to remain unaffected by PHMB, although no substantial growth of this pathogen was observed compared with negative control. These bacteriostatic findings concerning the PHMB additive are in line with previous reports where PHMB demonstrated effectiveness against the burden of a large variety of wound-colonizing microorganisms, including methicillin-resistant *S. aureus* (MRSA) [37]. However, no evidence of any resistance to PHMB was observed [38]. Although we tested important representatives of Gram-positive (*S. aureus*) and Gram-negative (*P. aeruginosa*) bacteria, examination of a much more comprehensive range of strains, particularly those Gram-negative pathogens colonizing wounds, is required to substantiate our findings. In addition, a study assessing the molecular biology of wound healing under the INTELIGEL smart dressing treatment with and without antibacterial agents is warranted.

There are several limitations to this study. The first one is the relatively small sample size; only one animal was used in each study, which increases the confounding and makes the statistical analysis impossible to perform. Burn injuries were created following established protocols by Moritz et al. [39] and adjusted and reproduced by Cuttle et al. [40]. The more superficial dermal partial-thickness burns in the first study and deeper ones in the second study (remaining in the dermal partial-thickness range) could be explained by the animals’ differences, proving its multifactorial origin as previously described [41]. The adjustments should be made for each animal to achieve the desirable burn depth [40]. Another difficulty was the lack of an objective assessment system on a macroscopic level. For further studies, a digital wound assessment software (DWAS) was warranted, combined with human expert opinion, to optimize the quantification of wound closure. A few Smart Bandage dressings were removed inappropriately; without liquefying the polymer using cold water. This technical issue is attributed chiefly to the lack of experience in wound care and poor familiarity with the polymer properties. The frequent change of dressings was done purely for documentation and photography reasons. Because of technical difficulties, ten out of 16 wounds (in the first study) were not appropriately stained for histological assessment and were excluded from the analysis, reducing the sample size to *n* = 6; these issues were eliminated in the second study, where *n* = 18. Ultimately, Smart Bandage+ (INTELIGELS Ltd. Hoshaya, Israel) was tested in vivo for its safety profile, while the efficacy profile was tested only in vitro. Further studies regarding the efficacy of Smart Bandage+ (INTELIGELS Ltd. Hoshaya, Israel) on actual inoculated wounds are warranted.

## 5. Conclusions

We conclude that owing to its histological and antimicrobial properties, Smart Bandage (INTELIGELS Ltd. Hoshaya, Israel) technology represented by hydrocolloids can complement commonly used hydrofiber dressings Aquacel^®^/Aquacel Ag^®^ and replace simple saline gauze dressings in the treatment of partial-thickness burns. Furthermore, hydrocolloid dressing developed by INTELIGELS Ltd. also allows the incorporation of a therapeutic agent and its delivery to the wound with the resultant improved wound healing that is evidenced by reduced inflammatory responses and shortened wound healing time at potentially lower costs. The results of this preclinical feasibility animal study favor further investigation in clinical settings to inform the utility of hydrocolloid technology (INTELIGELS Ltd. Hoshaya, Israel) in the clinical management of partial- thickness burns in humans.

## Figures and Tables

**Table 1:** Microbiological assessment of wounds.

Bacteria	Test Item	Average Recovery ± SD (Log_10_CFU per Sample)	Average Reduction ± SD (Log_10_CFU per Sample)	Statistical Significance (*p*-Value)
***P. aeruginosa***	Negative control	6.30 ± 0.03	N/A	N/A
	Smart Bandage+	6.11 ± 0.44	0.19 ± 0.44	N/A
***S. aureus***	Negative control	0.01 ± 0.11	N/A	N/A
	Smart Bandage+	0.00 ± 0.00	6.01 ± 0.00	*p* < 0.001

## Data Availability

Data are available upon request.

## Appendix A

**Table A1:** Semiquantitative Histological evaluation system.

Score Finding	0	1	2	3	4
**Superficial serocellular crusting or epidermal inflammation**	Normal epidermis	Rare serocellular crusts	Extensive serocellular crusts	Subcorneal pustules	Extensive inflammation on the surface
**Epidermal regeneration**	Complete epidermal regeneration	Complete epidermal regeneration + mild epidermal hyperplasia	Complete epidermal regeneration + moderate to marked epidermal hyperplasia	Incomplete epidermal regeneration	persistent ulcer/no epidermal regeneration
**Dermo-epidermal clefting**	None	Rare foci of separation	Occasional areas of separation of the dermo-epidermal junction	Formation of bullae	Extensive bullae formation or complete separation of the dermo-epidermal junction
**Granulation tissue**	Normal dermis	Collagen reaches tissue with mature fibrocytes	Granulation tissue with attenuated fibrocytes	Reactive granulation tissue with prominent blood vessels	Necrosis/early fibrosis with no granulation tissue

**Table A2:** Histological evaluation system—Dermal or panniculus inflammation.

Score Finding	0	1	2	3	4
**Dermal inflammation:**	None	Minimal	Mild	Moderate	Severe
**Granulocyte cell presence**	None	Minimal	Mild	Moderate	Severe
**Mononuclear cell (lymphocytes +plasma cells) presence**	None	Minimal	Mild	Moderate	Severe
**Macrophages and giant cell presence**	None	Minimal	Mild	Moderate	Severe
**Necrosis in the wound area**	Normal, no necrosis	Rare necrotic foci surrounded by macrophages	Minimal necrosis involving less than 25% of the wound area surrounded by macrophages Or Necrosis of epidermis adnexa and up to 10% upper dermis (depth of dermis)	Focal necrosis, involving 60% of the wound area Or Necrosis or sloughing of the epidermis. Adnexal necrosis. Necrosis of 10 to 25% depth of dermal necrosis	Complete wound area necrotic. Or Sloughing of the adnexal epidermis necrosis. More than 25% of death from dermal necrosis

**Table A3:** Average scoring of histological parameters based on Table A1 and Table A2.

Study	Group	Crust	Epidermal Regeneration	Cleft	Granulation Tissue	Dermis Inflammation	Mononuclear Cells	Necrosis in Wound Area	Total Average Score
**1**	Smart Bandage	1.5	2	0.5	2	1	1	0	8
	Aquacel^®^	2	1.5	0.5	1	1.5	0.5	0	7
	Simple saline gauze	2	2	1.5	3	0	0	0	8.5
**2**	Smart Bandage+	0.7	3.1	2.3	3.5	1.2	1.1	1.7	13.7
	Aquacel Ag^®^	0.7	2.8	2.7	3.7	1.3	1.0	2.0	14.2
	Simple saline gauze	1.3	4.0	2.7	4.0	3.0	2.0	3.3	20.3

## References

[1] WHO (2018). Burns. https://www.who.int/news-room/fact-sheets/detail/burns.

[2] Sadeghi-Bazargani H., Mohammadi R. (2012). Epidemiology of burns in Iran during the last decade (2000–2010): Review of literature and methodological considerations. Burns.

[3] Abdel-Sayed P., Hirt-Burri N., de Buys Roessingh A., Raffoul W., Applegate L.A. (2019). Evolution of biological bandages as first cover for burn patients. Adv. Wound Care.

[4] Rahmanian-Schwarz A., Beiderwieden A., Willkomm L.M., Amr A., Schaller H.E., Lotter O. (2011). A clinical evaluation of Biobrane(®) and Suprathel(®) in acute burns and reconstructive surgery. Burns.

[5] Schwarze H., Küntscher M., Uhlig C., Hierlemann H., Prantl L., Noack N., Hartmann B. (2007). Suprathel, a new skin substitute, in the management of donor sites of split-thickness skin grafts: Results of a clinical study. Burns.

[6] Dhivya S., Padma V.V., Santhini E. (2015). Wound dressings—A review. Biomedicine.

[7] Surowiecka A., Strużyna J., Winiarska A., Korzeniowski T. (2022). Hydrogels in burn wound management—A review. Gels.

[8] Chhabra S., Chhabra N., Kaur A., Gupta N. (2017). Wound healing concepts in clinical practice of OMFS. J. Maxillofac. Oral Surg..

[9] Derakhshandeh H., Kashaf S.S., Aghabaglou F., Ghanavati I.O., Tamayol A. (2018). Smart Bandages: The Future of wound care. Trends Biotechnol..

[10] Sosnik A., Cohn D. (2005). Reverse thermo-responsive poly(ethylene oxide) and poly(propylene oxide) multiblock copolymers. Biomaterials.

[11] Cohn D., Sosnik A., Levy A. (2003). Improved reverse thermo-responsive polymeric systems. Biomaterials.

[12] Cohn D., Sosnik A., Malal R., Zarka R., Garty S., Levy A. (2007). Chain extension as a strategy for the development of improved reverse thermo-responsive polymers. Polym. Adv. Technol..

[13] Cabodi M., Cross V.L., Qu Z., Havenstrite K.L., Schwartz S., Stroock A.D. (2007). An active wound dressing for controlled convective mass transfer with the wound bed. J. Biomed. Mater. Res. B Appl. Biomater..

[14] Shirakata Y., Tokumaru S., Yamasaki K., Sayama K., Hashimoto K. (2003). So-called biological dressing effects of cultured epidermal sheets are mediated by the production of EGF family, TGF-beta and VEGF. J. Dermatol. Sci..

[15] Chan E.S., Lam P.K., Liew C.T., Lau H.C., Yen R.S., King W.W. (2001). A new technique to resurface wounds with composite biocompatible epidermal graft and artificial skin. J. Trauma.

[16] Griffiths M., Ojeh N., Livingstone R., Price R., Navsaria H. (2004). Survival of Apligraf in acute human wounds. Tissue Eng..

[17] Yannas I., Burke J., Warpehoski M., Stasikelis P., Skrabut E.M., Orgill D.P., Giard D., Cooper S., Peppas N., Hoffman A., Ratner B. (1982). Design principles and preliminary clinical performance of an artificial skin. Biomaterials: Interfacial Phenomena and Applications.

[18] Winter G.D. (1962). Formation of the scab and the rate of epithelization of superficial wounds in the skin of the young domestic pig. Nature.

[19] Winter G.D., Scales J.T. (1963). Effect of air drying and dressings on the surface of a wound. Nature.

[20] Xu R., Xia H., He W., Li Z., Zhao J., Liu B., Wang Y., Lei Q., Kong Y., Bai Y. (2016). Controlled water vapor transmission rate promotes wound healing via wound re-epithelialization and contraction enhancement. Sci. Rep..

[21] Expert Working Group SEWG (2008). Wound exudate and the role of dressings. A consensus document. Int. Wound J..

[22] Dowsett C. (2012). Management of Wound Exudate.

[23] Wlaschin K.F., Ninkovic J., Griesgraber G.W., Colak Atan S., Young A.J., Pereira J.M., Solberg M.J., Smith G., Parks P.J., McNulty A.K. (2019). The impact of first-aid dressing design on healing of porcine partial thickness wounds. Wound Repair Regen..

[24] Shu W., Wang Y., Zhang X., Li C., Le H., Chang F. (2021). Functional hydrogel dressings for treatment of burn wounds. Front. Bioeng. Biotechnol..

[25] Harats M., Jaeger M., Kornhaber R.A., Haik J.M. (2016). AQUACEL® Ag burn glove and silver sulfadiazine for the treatment of partial thickness hand burns: A retrospective review. Indian J. Burns.

[26] Carter M.J., Tingley-Kelley K., Warriner R.A. (2010). Silver treatments and silver-impregnated dressings for the healing of leg wounds and ulcers: A systematic review and meta-analysis. J. Am. Acad. Dermatol..

[27] Yarboro D.D. (2013). A comparative study of the dressings silver sulfadiazine and Aquacel Ag in the management of superficial partial-thickness burns. Adv. Skin Wound Care.

[28] Sheckter C.C., Van Vliet M.M., Krishnan N.M., Garner W.L. (2014). Cost-effectiveness comparison between topical silver sulfadiazine and enclosed silver dressing for partial-thickness burn treatment. J. Burn Care Res..

[29] Caruso D.M., Foster K.N., Blome-Eberwein S.A., Twomey J.A., Herndon D.N., Luterman A., Silverstein P., Antimarino J.R., Bauer G.J. (2006). Randomized clinical study of hydrofiber dressing with silver or silver sulfadiazine in the management of partial thickness burns. J. Burn Care Res..

[30] Lo S.F., Chang C.J., Hu W.Y., Hayter M., Chang Y.T. (2009). The effectiveness of silver-releasing dressings in the management of non-healing chronic wounds: A meta-analysis. J. Clin. Nurs..

[31] Michaels J.A., Campbell B., King B., Palfreyman S.J., Shackley P., Stevenson M. (2009). Randomized controlled trial and cost-effectiveness analysis of silver-donating antimicrobial dressings for venous leg ulcers (VULCAN trial). Br. J. Surg..

[32] Eberlein T., Haemmerle G., Signer M., Gruber-Moesenbacher U., Traber J., Mittlboeck M., Abel M., Strohal R. (2012). Comparison of PHMB-containing dressing and silver dressings in patients with critically colonised or locally infected wounds. J. Wound Care.

[33] Piatkowski A., Drummer N., Andriessen A., Ulrich D., Pallua N. (2011). Randomized controlled single center study comparing a polyhexanide containing bio-cellulose dressing with silver sulfadiazine cream in partial-thickness dermal burns. Burns.

[34] de Mattos I.B., Holzer J.C.J., Tuca A.C., Groeber-Becker F., Funk M., Popp D., Mautner S., Birngruber T., Kamolz L.P. (2019). Uptake of PHMB in a bacterial nanocellulose based wound dressing: A feasible clinical procedure. Burns.

[35] Wild T., Bruckner M., Payrich M., Schwarz C., Eberlein T., Andriessen A. (2012). Eradication of Methicillin-Resistant Staphylococcus aureus in pressure ulcers comparing a polyhexanide containing cellulose dressing with polyhexanide swabs in a prospective randomized study. Adv. Ski. Wound Care.

[36] Wiegand C., Abel M., Ruth P., Hipler U.C. (2009). HaCaT keratinocytes in co-culture with Staphylococcus aureus can be protected from bacterial damage by polihexanide. Wound Repair Regen..

[37] Kirker K.R., Fisher S.T., James G.A., McGhee D., Shah C.B. (2009). Efficacy of polyhexamethylene biguanide-containing antimicrobial foam dressing against MRSA relative to standard foam dressing. Wounds.

[38] Gilliver S.C. (2009). PHMB: A well-tolerated antiseptic with no reported toxic effects. J. Wound Care..

[39] Moritz AR and Henriques F.C. (1947). Studies of Thermal Injury: II. The Relative Importance of Time and Surface Temperature in the Causation of Cutaneous Burns. Am. J. Pathol..

[40] Cuttle L., Kempf M., Phillips G.E., Mill J., Hayes M.T., Fraser J.F., Wang X.Q., Kimble R.M. (2006). A porcine deep dermal partial thickness burn model with hypertrophic scarring. Burns.

[41] Gibson A.L.F., Carney B.C., Cuttle L., Andrews C.J., Kowalczewski C.J., Liu A., Powell H.M., Stone R., Supp D.M., Singer A.J. (2021). Coming to Consensus: What Defines Deep Partial Thickness Burn Injuries in Porcine Models?. J. Burn Care Res..

